# Aluminum Foil vs. Gold Film: Cost-Effective Substrate in Sandwich SERS Immunoassays of Biomarkers Reveals Potential for Selectivity Improvement

**DOI:** 10.3390/ijms24065578

**Published:** 2023-03-14

**Authors:** Rostislav Bukasov, Alisher Sultangaziyev, Zhanar Kunushpayeva, Alisher Rapikov, Dina Dossym

**Affiliations:** Department of Chemistry, Nazarbayev University, Kabanbay Batyr Ave. 53, Astana 010000, Kazakhstan

**Keywords:** SERS, sandwich immunoassays, selectivity, nonspecific protein absorption, aluminum foil, silicon, LOD, MPT64, tuberculosis biomarker, nanotags

## Abstract

The first application of aluminum foil (Al F) as a low-cost/high-availability substrate for sandwich immunoassay using surface-enhanced Raman spectroscopy (SERS) is reported. Untreated and unmodified Al F and gold film are used as substrates for sandwich SERS immunoassay to detect tuberculosis biomarker MPT64 and human immunoglobulin (hIgG) in less than 24 h. The limits of detection (LODs) for tuberculosis (TB) biomarker MPT64 on Al foil, obtained with commercial antibodies, are about 1.8–1.9 ng/mL, which is comparable to the best LOD (2.1 ng/mL) reported in the literature for sandwich ELISA, made with fresh in-house antibodies. Not only is Al foil competitive with traditional SERS substrate gold for the sandwich SERS immunoassay in terms of LOD, which is in the range 18–30 pM or less than 1 pmol of human IgG, but it also has a large cost/availability advantage over gold film. Moreover, human IgG assays on Al foil and Si showed better selectivity (by about 30–70% on Al foil and at least eightfold on Si) and a nonspecific response to rat or rabbit IgG, in comparison to the selectivity in assays using gold film.

## 1. Introduction

There are numerous advantages of SERS as a very sensitive, label-free, humidity-independent, and rapid method, with great capability of multiplexing, which was discovered in the 1970s [1]. There is increasing interest in the scientific community for SERS as a versatile method for early medical diagnostics and reliable detection of major health threats in humans (e.g., cancer [2] and tuberculosis [3]) and animals [4]. For instance, sandwich immunoassays with SERS readout demonstrated an ultralow (1 pg/mL) limit of detection (LOD) for prostate-specific antigen [5]. Even lower detection limits were recently achieved in the detection of multiple viral antigens [6]. Multiple factors have an impact on sandwich SERS immunoassays, such as substrate composition, pH, temperature, and ionic strength of the buffer solutions where immunoreactions occur [7].

Currently, in the majority of sandwich SERS immunoassays, the substrate represents the primary component of the assay sequence that interacts with other components through chemical (with thiol-linker molecules of self-assembled monolayer (SAM)), physical (via electrostatic and van der Waals forces), and plasmonic (with ERL gold core and reporter molecules) interactions. Most sandwich SERS immunoassays use gold film as the solid substrate, such as those reported by the research groups of Chang [8], Choo [9], Chung [10], Driskell [11], Krasnslobotsev [12], Lipert [2], Porter [4], and Trau [13]. Some reports exist on the application of other solid substrates in SERS sandwich immunoassays. For instance, an assay was introduced where the capture antibody is physisorbed on the nitrocellulose membrane (NCM), but this assay failed to achieve similar performance to typical assays using gold in terms of sensitivity and LOD (LOD of 1.25 ng for antigen on NCM), being lower by 2–4 orders of magnitude [14].

Overall, to the best of our knowledge, hardly any studies exist comparing various substrates in sandwich SERS immunoassays. Typically, SERS sandwich assays are conducted using gold film modified with SAM of DSP, DSU, or other linkers with a succinimidyl group that binds to capture proteins (e.g., a-hIgG) [15]. However, Porter’s group recently showed that the efficiency of linkers with the succinimidyl group is nearly negligible, due to the competing reaction of group hydrolysis, which may be threefold higher than for aminolysis [16]. As ultrasensitive SERS sandwich immunoassays move closer toward their application in medical diagnostics in third-world countries (e.g., for tuberculosis [3]), options for reducing assay cost and time, without significantly compromising assay sensitivity, become even more relevant.

One of these options is the application of a less expensive substrate metal or other materials; another option is the elimination of substrate modification using DSP or any other specific linker, instead relying on nonspecific adsorption for immobilization of the capture antibody or antigen. A combination of these two options is clearly desirable.

In this article, we investigate the potential of Al foil, one of the most available metallic materials, as a SERS sandwich substrate. Although its cost is much lower than that of gold film, Al foil cannot strongly adsorb mercaptans and other sulfur-containing compounds in comparison to gold film. Furthermore, in field/clinical analysis, the gold film may not be “freshly prepared”, but instead used months after preparation in another location. Therefore, it may often be contaminated, necessitating plasma or piranha solution cleaning before use in the immunoassay, which can be problematic and inconvenient.

There are increasing numbers of publications on SERS-based immunoassays conducted using various, non-noble metal flat film substrates, including paper-based, bimetallic, and composite nanoparticle-based substrates [17,18,19,20,21]. However, some of those substrates are relatively expensive and complicated in terms of preparation and/or synthesis.

Aluminum and silicon may represent cost-effective and robust alternatives to gold or silver films/nanostructures. Several plasmonic applications of Al nanoparticles in the UV range, including both experimental reports and theoretical predictions, have been reported. Examples of these applications include deep UV (DUV) SERS, DUV tip-enhanced SERS, and surface-enhanced fluorescence [22,23,24]. Recent publications have reported the application of Al alloy and Al foil as substrates for SERS with visible excitation [25,26,27]. Several other reports also exist on the efficient application of Al foil, sometimes performing on par with gold film, as a substrate for surface-enhanced spectroscopies, including surface-enhanced fluorescence [28,29,30] and SERS [31,32].

Silicon nanohybrid-based SERS substrates, such as gold/silver nanoparticle (NP)-decorated silicon nanowires and Au/Ag NP-decorated silicon wafers (AuNP@Si), have been reported for the detection of several chemical and biological compounds [33]. Silicon wafer has been reported as a substrate for use in the very sensitive SERS aptasensor for the detection of ricin B toxin [34]. Recently, Kunushpayeva et al. reported the first application of silicon as a substrate for a SERS sandwich immunoassay, where capture antibodies were attached directly to the silicon wafer [35].

Hereinafter, we report the first application of Al foil, covered with several nanometers of oxide film [24], as a substrate for SERS sandwich immunoassays with excitation in the visible light range (633 nm). MPT64 is an immunogenic protein, which is highly specific to *Mycobacterium tuberculosis* [36]. According to the World Health Organization, tuberculosis (TB) claimed 1.8 million deaths worldwide while an estimated 49 million lives were saved through TB diagnosis and treatment between 2000 and 2015 [37]. In this paper, for the first time, we report the SERS immunoassay detection of tuberculosis biomarker protein MPT64 using aluminum foil, and we compare its performance to gold film. The detection of MPT64 using a sandwich ELISA with freshly made in-house antibodies, is reported [38]. We demonstrate that the reported sensitivity of SERS can be matched or improved by the application of a low-cost /high-availability substrate, Al foil, even when commercial antibodies are used.

The nonspecific adsorption/binding of proteins, particularly those with similar properties to the analyte (e.g., IgG), significantly hinders the sensitivity and selectivity/specificity of sandwich immunoassays [39]. Therefore, the selectivity of SERS sandwich immunoassays for human IgG is compared in terms of a specific response to human IgG with a nonspecific response to other IgG (e.g., rat) on three substrates: gold film, aluminum foil, and silicon. This assay is followed by an SEM characterization of the substrates, as well as a discussion of the results.

## 2. Results and Discussion

### 2.1. Raman Spectra, Calibrations, and LODs for Assays on Al Foil vs. Simultaneous Assays on Gold Film

**Figure 1:**
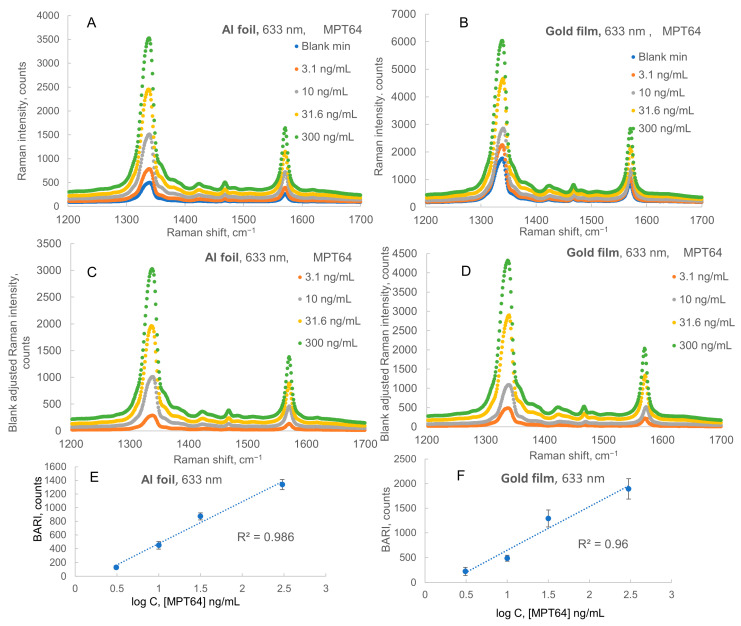
Raman spectra and calibration for sandwich immunoassay of MPT64 on Al foil and gold film with 633 nm excitation. (**A**,**B**) Spectra of Raman intensity on Al foil and Au film, respectively. (**C**,**D**) Blank-adjusted Raman intensity (BARI, counts) on Al foil and Au film, respectively. (**E**,**F**) Logarithmic calibration plots (BARI vs. decimal logarithm of MPT 64 biomarker concentration (ng/mL)) on the same substrates, respectively, with correlation coefficients R^2^ for each plot.

**Figure 2:**
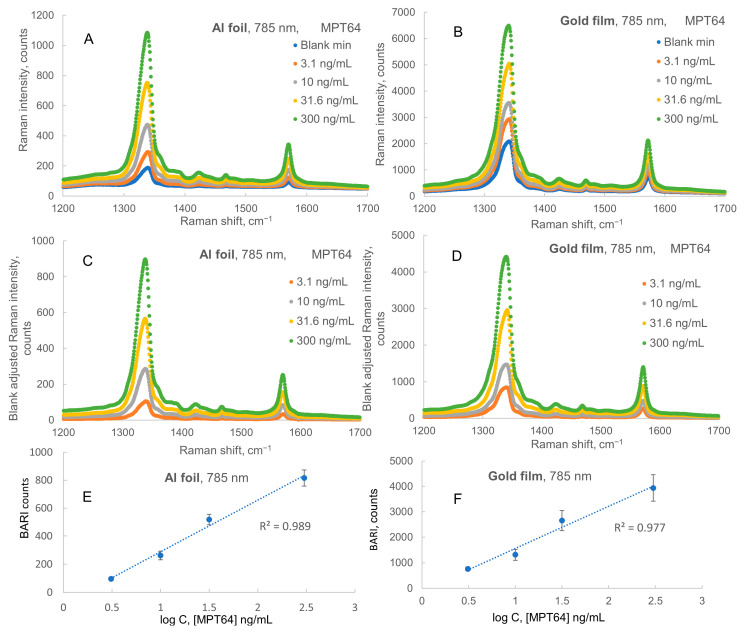
Raman spectra and calibration plots for sandwich immunoassay of MPT64 on Al foil and gold film with 785 nm excitation. (**A**,**B**) Spectra of Raman intensity on Al foil and Au film, respectively. (**C**,**D**) Blank-adjusted Raman intensity on Al foil and Au film, respectively. (**E**,**F**) Logarithmic calibration plots on the same substrates, respectively.

The averaged normalized Raman spectra for 633 nm and 785 nm laser excitations and the calibration plot for the SERS immunoassay of MPT64 are shown in Figure 1 and Figure 2, respectively. In this assay, 50 nm diameter nanoparticles from Sigma Aldrich were used for the preparation of nanotags. Figure 1 and Figure 2 show that Raman intensity remained about the same when we compare results obtained with the 633 nm and 785 nm lasers on gold film substrate. We found that LODs calculated from the plots using logarithm trendlines in the 3–300 ng/mL MPT64 concentration range were 1.9 ng/mL on Al foil and 2.9 ng/mL on gold film when 633 nm laser excitation was used. When we used 785 nm laser excitation, we obtained slightly improved LODs: 1.8 ng/mL on Al foil and 1.3 ng/mL on gold film. Therefore, overall, both substrates had comparable performance in terms of LOD: slightly better LOD on Al foil vs. on gold at 633 nm excitation, but slightly better LOD on gold vs. on Al foil at 785 nm excitation. All the data used for calibration of LODs are included in Supplementary Table S1. Logarithmic trends worked a bit better on Al foil when compared to gold film for the average of two excitation wavelengths (R^2^ = 0.988 on Al foil and R^2^ = 0.968 on gold film). The slopes on calibration plots were higher on gold than on Al foil, but blank Raman intensities and standard deviations of the blank were significantly lower on Al foil; therefore, on average, the ratio of the standard deviation of the blank to the slope of the calibration plot was slightly lower on Al foil in comparison to gold film. Overall, the results demonstrated a slightly lower LOD for the SERS sandwich immunoassay of MPT64 using Al foil (1.8 or 1.9 ng/mL or just 54 or 57 pg of the biomarker) with commercial antibodies, which were produced several months before the assay date, in comparison to the LOD reported for sandwich ELISA using custom-made “fresh” antibodies of 2.0 ng/mL [38]. Since we used non-lyophilized antibodies and antigens particularly sensitive to storage conditions (−20 °C or less recommended), with a process of delivery to NU in Kazakhstan a few weeks long, including customs clearance, this relatively high assay sensitivity showed the significant robustness of the SERS sandwich immunoassay method using both gold and Al foil substrates.

Figure 1 and Figure 2 show that Raman intensities remained about the same when we compare results obtained using the 633 nm and 785 nm lasers on the gold film substrate, whereas they decreased from 633 nm to 785 nm laser excitation for the results measured on Al foil. Therefore, we selected a 633 nm He–Ne laser for subsequent measurements, i.e., comparative immunoassays of human IgG as a model antigen on three substrates and assessment of assay selectivity for human IgG vs. rat IgG on these three substrates.

**Figure 3:**
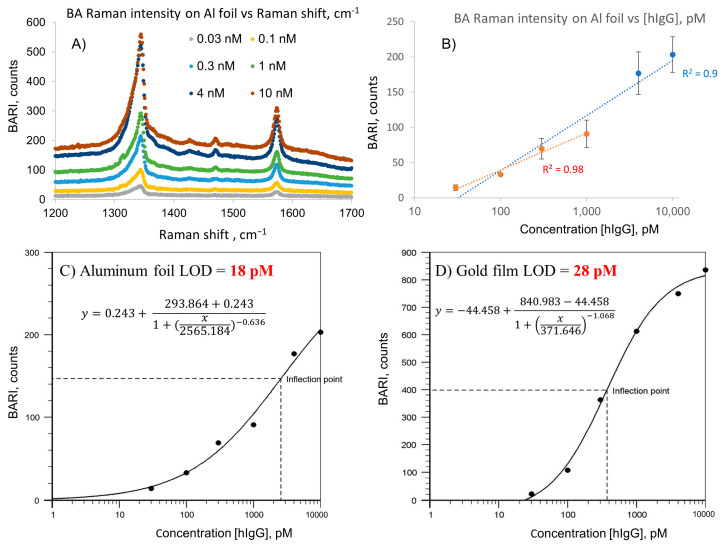
SERS spectra and logarithmic calibration plot of sandwich SERS immunoassays of human IgG on Al foil (**A**,**B**). Four-parameter logistic nonlinear regression analysis calibration plots of the same-day assay of human IgG using Al foil (**C**) and on gold film (**D**). The latter was adopted from the Supplementary Materials of Kunushpayeva et al. [35]. NBARI is the normalized blank-adjusted Raman intensity. R^2^ = 0.99 for Al foil and 0.98 for gold film.

We performed a comparative assay of human IgG on gold, silicon, and Al foil, using practically the same assay parameters as those in the MPT64 assay; however, for nanotag preparation, instead of 50 nm nanoparticles used in the MPT64 assay, we used larger commercial nanoparticles of 60 nm average diameter from the same producer (suspension in PBS from Sigma Aldrich), since there are a few reports in the literature of SERS immunoassays using this size of nanoparticles (e.g., by Porter’s research group [40]). The averaged normalized Raman spectra and calibration plot for the SERS immunoassay of human IgG on Al foil are shown in Figure 3**.** The LOD of 30 pM was calculated from the trendline of signal vs. logarithm of concentration (R^2^ = 0.98) on plot B of this figure for the assay using Al foil in the 30–1000 pM human IgG concentration range. The LOD of 37 pM was calculated from the same trendline (R^2^ = 0.96) in the simultaneous assay of human IgG using gold film described in Kunushpayeva et al. in the same range [35]. Data for the calculation of both LODs (Al foil and gold) are shown in Supplementary Table S2. We also used four-parameter logistic nonlinear regression analysis as a common tool for calibration in biodetection techniques such as ELISA [41]. The results are shown in Figure 3C,D, where we obtained an even better R^2^ = 0.99 for the same data from the assay using Al foil and a better LOD of 18 pM for Al foil in comparison to 28 pM for gold, calculated in the same range of concentrations from six data points.

The common tendency for the immunoassays of MPT64 and hIgG is a decrease in Raman intensity for the same order of substrates: gold film, Al foil, and Si wafer. However, we previously reported that the LOD for gold was numerically higher than that for silicon [35]. Here, we observe that the LOD for gold is also numerically higher (worse) than that for Al, while the R^2^ coefficients for calibration on Al foil are closer to one (better) than those on gold film. Tables S1 and S2 in Supplementary Information show the data used in calculation of LODs for detection of MPT64 and human IgG, respectively.

### 2.2. Selectivity Assay of Human IgG vs. Rat IgG on Three Substrates: Al Foil Tape, Gold Film, and Silicon

**Figure 4:**
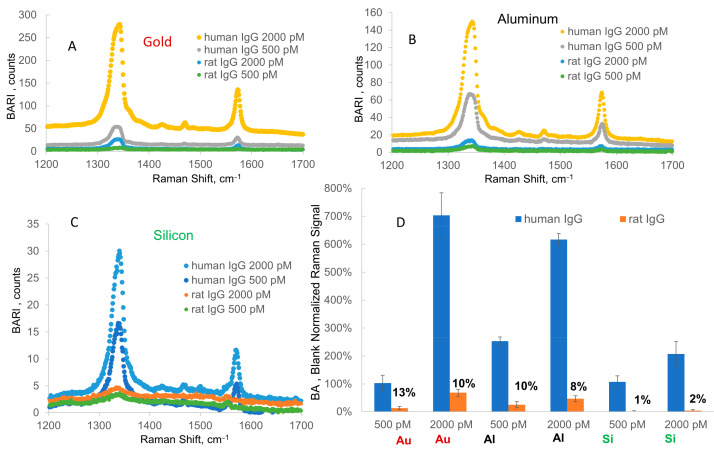
SERS blank-adjusted spectra in selectivity assay: human and rat IgG response on (**A**) hold, (**B**) Al tape, and (**C**) Si wafer. (**D**) Blank-adjusted blank-normalized Raman intensity/signal for each substrate (IgG concentrations of 500 and 2000 pM). Here, the response/signal for each concentration is normalized to the response for a blank on each substrate (13%, 10%, etc.), showing the ratios of nonspecific (rat IgG) to specific (human IgG) signals for each concentration using gold, aluminum, and silicon.

In order to assess the selectivity in the detection of human IgG, we performed a comparative assay of detection of human IgG vs. rat IgG on three substrates: gold film, Al foil tape, and silicon wafer. In this assay, we used human IgG or rat IgG as antigens (analytes), and all other assay parameters were identical for all antigens and all substrates. The capture antibody on each substrate and on the ERLs (nanotags) was the same: anti-human IgG. All other assay parameters were absolutely identical for both antigens and all three substrates. In this assay, we compared the specific response of the assay to human IgG and the nonspecific response of the assay to the same concentration of rat IgG (500 and 2000 pM). The results of this assay are shown as three blank-adjusted Raman spectra and the bar graph in Figure 4. The bar graph in Figure 4D shows the blank-adjusted Raman intensity normalized to the blank signal (100%) specific to each substrate. The visual outcome of this bar graph is a ratio of the specific signal from human IgG (blue bar) to the nonspecific signal (light-brown bar). Obviously, a higher ratio denotes greater specificity of the assay for human IgG vs. rat IgG. We normalized the nonspecific signal by the specific signal from binding of human IgG (taken as 100%). Those normalized nonspecific signals are shown in Figure 4D for each concentration on each substrate (13%, 10%, etc.). Apparently, a lower number denotes that a lower nonspecific signal was observed, indicating a greater specificity of the assay for hIgG. Here, the assay on Al foil tape showed a less nonspecific signal (relative to specific signal) than that on gold film, which was the case for both tested antigen concentrations. Indeed, comparing relative nonspecific signals for Al and gold, we can see that 9.9% (Al) < 12.6% (Au) for the 500 pM concentration and 7.6% (Al) < 9.7% (Au) for the 2000 pM concentration. Overall, for these two concentrations, the relative nonspecific signal was higher on gold than on Al foil by 27% on average. However, the assay on silicon demonstrated a much bigger advantage in terms of selectivity, whereby the nonspecific signal from rat IgG binding was only 1–2% when normalized to the signal of human IgG for each concentration. Overall, for these two concentrations, the relative nonspecific signal was higher on gold than on silicon wafer Al foil by a factor of eight (or 843%) on average. Thus, in raw gold, aluminum foil, and silicon, the nonspecific signal from rat IgG binding apparently decreases in both relative and absolute terms, with the blank signal also decreasing in the same row.

Results of a similar assay on the same three substrates but with three antigens (human, rat, and rabbit IgG) are included in Supplementary Table S3 and Supplementary Figure S3. In this assay, we used 60 nm diameter nanoparticles as the only potentially significant difference from the assay described in Figure 4. Here, the blank-adjusted nonspecific signal/response of rat and rabbit IgG was normalized to a specific response for the same concentration of hIgG. According to the table in this figure, the assay specificity for Al foil relative to gold was 1.5–2.2 times higher (1.8 on average). For instance, for the same 0.04 nM rabbit IgG concentration, the relative nonspecific response was 3.8% on Al and 8.6% on gold. However, the specificity on Si was about 18 times higher, whereby the relative nonspecific response of 0.5 nM rabbit IgG was 0.5% on Si and 9.0% on gold. The nonspecific signals after subtraction of the blank signal for the 0.04 nM antigen concentration were positive but very small (within uncertainty); therefore, we only calculated relative nonspecific signals for the concentration of 500 pM. Overall, both assays demonstrated the same trend of increasing specificity or decreasing nonspecific response when the assay substrate was changed from gold to Al foil, and this trend was even more evident when the assay substrate was changed to silicon.

We conducted another human/rat IgG assay using the same conditions and materials as in the assay for MPT64, and we characterized the results on two substrates, gold film and Al foil, using SEM. We did not include silicon in the SEM characterizations since comparative characterization of the simultaneous assay on gold and silicon using AFM was previously reported in Kunushpayeva et al. [35].

### 2.3. SEM Characterization of Immunoassay of Human IgG on Gold and Al Foil

**Figure 5:**
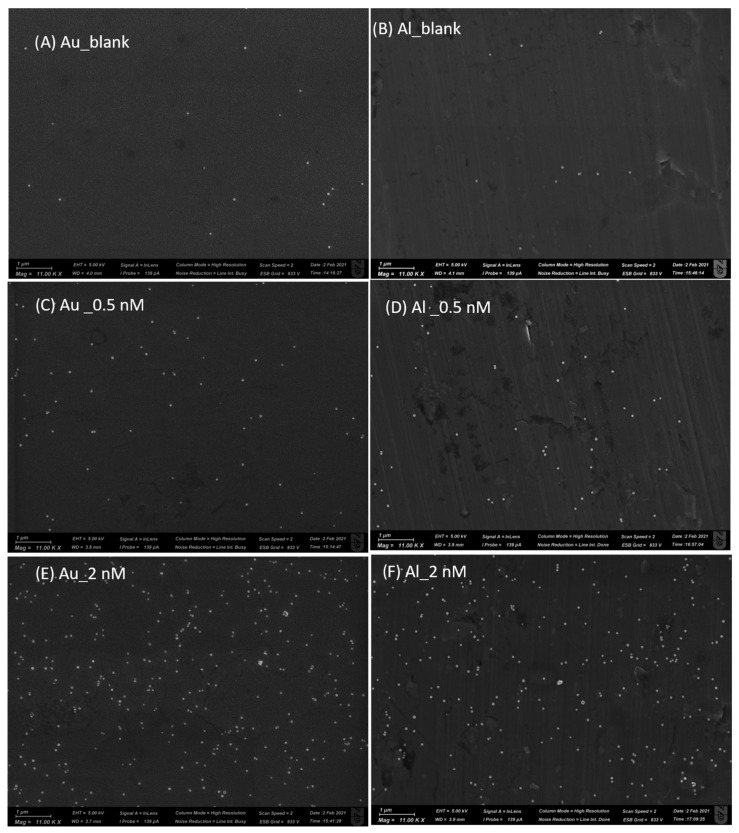
Representative SEM images of sandwich immunoassay on gold and Al tape with human IgG concentrations of 0 (blank), 0.5, and 2 nM.

We provide representative SEM images of this characterization in Figure 5. Figure S4 in Supplementary Information shows representative SEM images for 0.04 nM human IgG samples on gold and on Al tape. Table 1 shows the parameters calculated from this characterization for eight samples: four samples on each substrate (blank, 0.04 nM, 0.5 nM, and 2 nM human IgG samples).

A total of 3649 nanotags were counted on 32 SEM maps (four on each sample and 16 on each substrate), including 2751 nanotags on gold film and 898 nanotags on Al foil. These nanotags were classified by aggregation (single, dimers, trimers, or oligomers), and the number of aggregation states was calculated for each sample.

**Figure 6:**
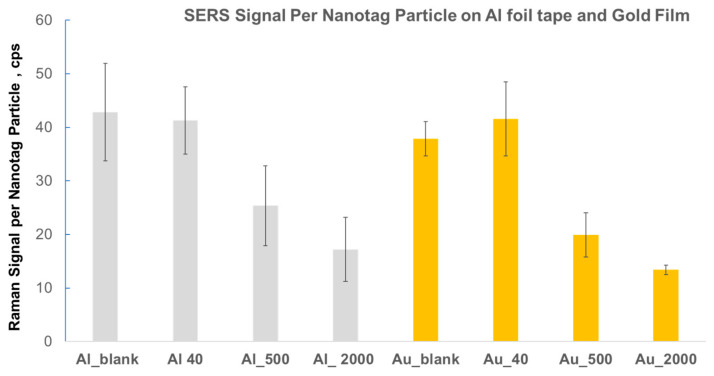
Raman signals per nanotag particle (for laser beam area: 1 µm^2^) calculated from SEM characterization of human IgG assay on gold film vs. Al foil tape. As an example of the nomenclature, Au_40 shows the 40 pM concentration of human IgG on gold film.

As expected, the number of nanoparticles per area increased when the concentration of antigen (human IgG) increased. Table 1 demonstrates that overall aggregation profiles on both substrates were somewhat similar: on average, the majority of nanotags on gold (76%) and Al foil (76%) were singles (not aggregated), along with 14–15% of nanotags aggregated in dimers on both substrates, and 2–3% of nanotags aggregated in trimers on both substrates. We plot the ratio of Raman signal to the number of ERLs per µm^2^ in Figure 6. The SERS intensity per nanotag particle is proportional to the ratio of Raman signal to number NP/area; when the focused laser beam area is 1 µm^2^, the two ratios are identical. In any case, the laser beam cross-section/area remained constant for both substrates and all characterized samples; thus, this ratio can be effectively used for the comparison of SERS intensities per nanotag on different substrates at different concentrations. Figure 6 shows comparable Raman intensities per nanotag on gold film and Al foil tape.

This figure also demonstrates a general decreasing trend in Raman signal per nanoparticle for the SERS assay on both substrates (Al foil and gold film). A similar trend was previously reported and discussed for assays on silicon and gold film [35]. A possible explanation for this trend is a decrease in the extinction efficiency of nanoparticles, which is somewhat proportional to SERS enhancement when the surface concentration of nanoparticles increases (number NPs/area), as observed by Bukasov et al. [42]. The only exception in Figure 6 is a slight increase in signal per nanotag from 38 to 45 cps/NP from the blank to 40 pM hIgG sample; however, this increase can be explained by a noticeable increase in the fraction of dimers from 9% to 14% and, to a lesser extent, by an increase in the fraction of trimers from 0% to 3%. SERS enhancement factors (EFs) for gold nanoparticle dimers on gold film were approximately 1.2–1.4 times higher than those for single nanoparticles measured for the same samples, while EFs for trimers were about 40–80% higher than EFs for singles, according to Sergiienko et al. [43]. Therefore, with an increasing fraction of dimers and trimers, we would expect an increasing SERS signal per nanoparticle, particularly when the number of nanotags per area does not change significantly (change from 0.40 to 0.41 NP/µm^2^ or within experimental uncertainty).

### 2.4. Discussion of Nonspecific Binding and Selectivity

As shown in Table 1, the surface concentration of ERLs in the blank on gold (0.402 NP/µm^2^) was significant, being about 70–80% higher than that on Al foil (0.226). This observation indicates that nonspecific adsorption of ERLs (nanotags), modified with capture antibodies to the substrate modified with the same capture antibodies, was significantly higher when the substrate was gold relative to the case when the substrate was Al foil.

Indeed, one of the components contributing to this nonspecific interaction is Van der Waals forces that are proportional to the Hamaker constant for the metal. The Hamaker constant for gold is higher than for Al by approximately 20–25%, as calculated from optical data [44]. The oxide layer on the surface of the Al foil probably further reduces the Hamaker constant for these Van der Waals interactions in comparison to gold. The Van der Waals interactions between gold nanospheres and silicon substrate are also significantly weaker than those between gold nanospheres and gold substrate, as demonstrated by the lower Lifshitz–van der Waals constant for Au–Si (5.32 eV) than Au–Au (9.85 eV) when both interactions are measured in water, as reported by Ahmadi [45].

However, the major driver for protein adsorption to silicon is likely a negative charge on the silicon surface, which is formed due to silanol group ionization in the formed native oxide layer on Si at physiological pH [46]. This factor can explain the smaller difference (45%) for a surface concentration of the 60 nm diameter ERL/nanotag between blank samples on gold (0.55 NP/µm^2^) and silicon (0.38 NP/µm^2^), as reported in Kunushpayeva et al. [35], compared to the same difference between ERL surface concentrations for blank samples on gold and Al foil (78%).

In the case of aluminum vs. gold substrates, a more important factor than Van der Waals interactions contributing to the stronger binding of hIgG and/or hIgG to gold rather than aluminum is probably the higher energy of adsorption of sulfur on gold (e.g., 0.7 eV for cysteine/Au) in comparison to that on aluminum (e.g., 0.5 eV for thiophene/Al (111)), which reduces the contribution of sulfur–metal interaction to protein binding [47,48]. The reason for this is the formation of a gold–sulfur bond, which has intermediate energy between van der Waals and covalent bonding. This bond can be formed by the SH group of cysteine as at least one unmasked amino acid is present in human or other IgG molecules [49].

Therefore the advantage of lower nonspecific binding for immunoassays on Al foil and Si relative to gold film may explain the observed advantage in selectivity, an important analytical parameter, which is rarely evaluated in sandwich SERS immunoassays [50,51,52].

The mechanism of this advantage underlying selectivity/specificity has at least two components: lower Van der Waals interactions with Al foil and silicon in comparison with gold, and lower binding affinity of sulfur-containing amino acids to Al foil or silicon when compared to gold, as described above.

## 3. Materials and Methods

### 3.1. Materials

Nanoparticle suspensions (50 and 60 nm diameter) in PBS and all chemicals used in the assay preparation were purchased from Sigma Aldrich, Gillingham, UK; including 4-nitrobenzenethiol (NBT), anti-human IgG antibodies dissolved in PBS, human IgG, BSA (bovine serum albumin), casein, and Tween-20. The only exceptions were anti-MPT64 monoclonal antibodies and MPT64 protein (antigen), which were purchased from BBI Solutions, Portland, OR, USA, and Enogen, Cambridge, UK respectively. Microscope slides coated with gold film (100 nm thick layer, 99.9% purity) over a Cr (2–3 nm) layer were purchased from EFM Co., Salt Lake, UT, USA.

### 3.2. Assay Procedure

The assay procedure had the same basic steps as those by Porter’s group [15]. However, the current procedure applied no linker molecule for the capture antibody and followed the sequence described in Kunushpayeva et al. [35]. A general step-by-step scheme for the immunoassay, including the preparation of ERLs and modification of the substrate, is shown in Supplementary Figure S1, adopted from Kunushpayeva et al. with minor changes [35].

The assay procedure is illustrated in Supplementary Figures S1 and S2 and described below. After 5 mm diameter holes were punched in parafilm, this parafilm was applied to the metal film surface or silicon wafer and heated for 30–60 s at 70–80 °C to melt the parafilm and encourage its adherence to the film. The assay addresses spots, encircled by the parafilm, on the substrate fabricated using unmodified Al foil, gold, or silicon wafers. All assay reactions, each about several hours long, were performed in mini wet champers: inverted Petri dishes, saturated with moisture from water droplets in between samples. A picture of the typical assay is also included in the Supplementary Materials. The spots were incubated in a 30 µL solution of 20 µg/mL anti-MPT64 monoclonal antibodies or in a 30 µL solution of 20 µg/mL anti-human IgG antibodies dissolved in PBS (phosphate-buffered saline, pH = 7.4) for 3 or 4 h to produce the physisorbed capture antibody layer.

Next, all spots were rinsed thrice with PBST (0.1% Tween-20 in PBS). Then, all addresses are drop-casted with 30 µL of blocking agent (casein) for 3 or 4 h. Next, after another rinsing, they were covered with 25 µL droplets of analyte (MPT64 or human IgG solutions in PBS) at various concentrations for 3 or 4 h. Then, 30 µL aliquots of extrinsic Raman labels (ERLs) or nanotag suspensions were drop-casted on each spot of 5 mm diameter. Exposure to ERLs constituted 6 or 10 h of the 17 or 24 h total assay time, respectively.

ERLs were prepared from 50 or 60 nm gold nanoparticles by modification with 4-nitrobenzenethiol (NBT), instead of 5,5-dithiobis(succinimidyl-2-nitrobenzoate (DSNB), for 2 h, followed by centrifugation/resuspension in borate buffer 2mM (BB), introduction of the binding capture antibody (3.5 h), blocking with BSA (3.5 h), and three cycles of centrifugation/resuspension in BB following the sequence described in the literature [15]. When the total assay time was 17 h, binding antibody, blocking, and binding antigen steps were 3 h each; however, when the total assay time was 24 h, they were 4 h each, while the time for modification with NBT and centrifugation/resuspension remained the same.

Due to the proven low yield of aminolysis, we did not use linkers (dithiobis(succinimidyl propionate) or DSP and DSNB) in the assay, saving time and money during preparation. Overall, our assay procedure is significantly shorter, usually completed within 17 or 24 h, instead of 2–2.5 days for procedures reported in the literature [15]. Lastly, after triple rinsing and drying of the samples, they were ready for Raman measurements.

### 3.3. Measurements and Data Analysis

Raman emission intensity was measured using a LabRAM HR Evolution microscope system from HORIBA (Kyoto, Japan), with a He–Ne 632.8 nm (Melles Griot, Voisins-le-Bretonneux, France) laser or 785 nm diode laser (Sacher, Marburg, Germany), using a thermoelectrically cooled CCD detector and 10× objective. This objective was selected as a better alternative to the 50× objective because the spot appears 25–100 times larger, resulting in a substantially lower sampling error [40].

Raman spectra in the 1000–1600 cm^−1^ range were recorded with the following parameters: map size of 10 × 10 datapoints, step size of 100 µm, power on sample of about 5 mW, and typical integration time for each data point of 2 s. Usually, four maps were collected for each standard or blank on the same day and averaged.

The background-adjusted Raman intensities, corresponding to the characteristic Raman peak of NO_2_ stretching in the NBT molecule at about 1336 cm^−1^, were calculated for all samples. Then, the intensity of the blank was subtracted from the intensity for each standard. Calibration plots of normalized background-adjusted Raman intensity vs. the log of antigen concentration were established for each SERS immunoassay on a specified substrate. The limit of detection (LOD) was calculated as the concentration where the Raman intensity was equal to three standard deviations of the signal of the blank, according to the trendline on the calibration plot, using the formula shown in the Supplementary Materials. We obtained SEM maps using a Zeiss Crossbeam 540 SEM (Germany), with 4 kV voltage at 11,000× magnification.

## 4. Conclusions

This study proposed Al foil as a cost-effective, stable, and sensitive substrate for sandwich SERS immunoassays for a variety of applications in diagnostics and biodetection. For instance, just 50–60 pg of TB biomarker MPT64 or human IgG could be detected on unmodified Al foil. Both 785 and 633 nm lasers can be used in sandwich immunoassays with SERS detection on Al foil, as demonstrated using MPT64. Some disadvantages in Raman signal intensity of the hIgG assay on Al foil relative to the assay on gold are typically compensated for by the higher standard deviation of the signal on gold and the higher ability of Al foil to discriminate between the binding of complementary and noncomplementary IgGs, resulting in a higher specificity in human IgG detection on Al compared to gold.

Unmodified Al foil as a substrate in SERS sandwich immunoassays demonstrated a similar or better (numerically lower) LOD in comparison to gold film. For applications where the signal-to-instrument noise ratio is sufficiently high and the specificity/selectivity of the assay is important, because of its large and significant relative advantage in selectivity compared to gold (1.3–2-fold for Al foil and eightfold for silicon), Al foil or silicon can be used as substitutes for gold in the SERS immunoassay.

Overall, there was an apparent tradeoff between SERS signal intensity and selectivity for the detection of hIgG on the three substrates (Au, Al foil, and Si), with Al foil presenting intermediate. Therefore, considering its unbeatable advantage over gold film (and, to a lesser extent, over Si wafer) in terms of cost and availability, as well as its advantage in selectivity, we can suggest that Al foil as a preferable substrate in many SERS sandwich immunoassay applications. Insights into the effect of substrate on SERS sandwich assay performance can help in the design of new, more efficient SERS assays for various medical and biological sensing applications.

## Figures and Tables

**Table 1:** Results of SEM characterization of hIgG assay on gold film and Al foil.

		Number NP/Area, NP/µm^2^	Raman Intensity, cps	Signal/ (#NP/area)	% Single NPs	% Dimer NPs	% Trimer NPs	% Oligo NPs
Gold	Blank	0.40	15.2	37.8	80	9	0	10
	40 pM	0.41	18.4	44.8	72	14	3	7
	500 pM	1.79	35.6	19.9	74	18	3	4
	2000 pM	10.1	134.7	13.4	76	15	4	4
Al Foil	Blank	0.23	9.69	42.8	76	24	0	0
	40 pM	0.36	14.7	41.3	78	10	0	12
	500 pM	1.49	37.8	25.4	73	13	3	11
	2000 pM	4.94	85.2	17.2	72	12	5	11

## Data Availability

Not applicable.

## Supplementary Materials

The following supporting information can be downloaded at https://www.mdpi.com/article/10.3390/ijms24065578/s1. Reference [35] is cited in Supplementary Materials.
