# Supplementary material for: Aluminum Foil vs. Gold Film: Cost-Effective Substrate in Sandwich SERS Immunoassays of Biomarkers Reveals Potential for Selectivity Improvement
Source: Int J Mol Sci. 2023 Mar 14;24(6):5578. doi: 10.3390/ijms24065578 (PMC10051902; doi:10.3390/ijms24065578)
Supplement: Supplementary file 1 [file ijms-24-05578-s001.zip › ijms-2237220-supplementary.pdf]

<u>**Supplementary Information for**</u>

# Aluminum Foil vs. Gold Film: Cost-Effective Substrate in Sandwich SERS Immunoassays of Biomarkers Reveals Potential for Selectivity Improvement

Rostislav Bukasov, Alisher Sultangaziyev , Zhanar Kunushpayeva, Alisher Rapikov, Dina Dossym

Chemistry Department, SSH, Nazarbayev University, Nur-Sultan, 010000, Kazakhstan;

e-mail: rostislav.bukasov@nu.edu.kz

## Sandwich Immunoassay Preparation

## The scheme of the assay is shown on Figure S1 below

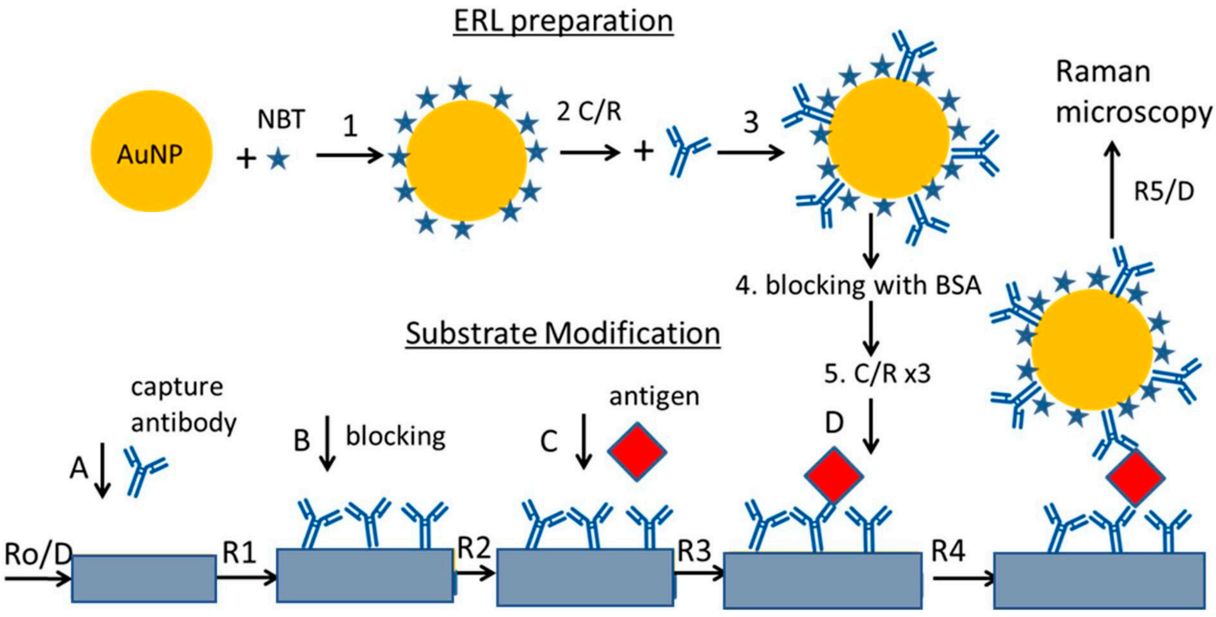


Figure S1 Scheme of Sandwich SERS immunoassay on Al foil substrate. Steps 1-4 of ERL preparation are shown above. A,B,C,D steps in substrate modification, where 25µL of antibody, antigen, Starting Block and ERL suspension, respectively are dropcasted on each address , R/D is rinsing /drying of the substrates, C/R is centrifuged /resuspended in preparation of ERL suspension. TEM image of a dimer from of 60 nm AuNPs (Sigma Aldrich), which were used in ERL preparation, illustrates their non-ideal (non-spherical) shape. The figure is adopted with minor modification from Kunushpayeva, et al [1], under Creative Commons License.

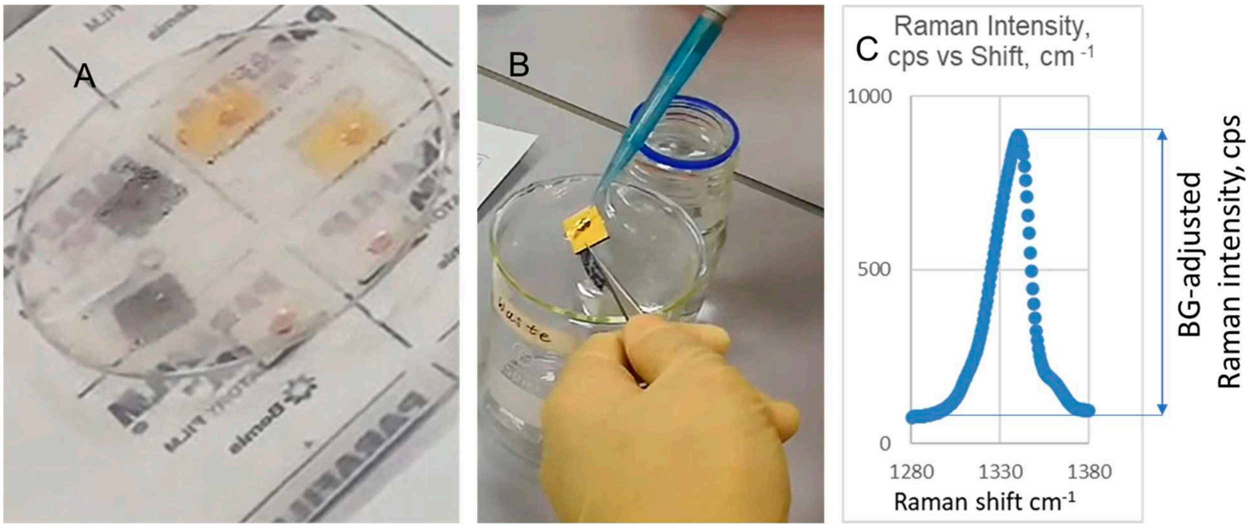


Figure S2 Sandwich SERS immunoassay: A. Samples on 3 substrates are kept in mini wet chamber: inverted Pettri dish on parafilm saturated with moisture to prevent drying; B. Process of rinsing with PBS buffer; C. Background adjusted Raman intensity

Substrates are 100 nm thick commercial gold film evaporated on microscope slides, or monocrystalline Silicon wafers. The surfaces of all substrates (average size 13 x 13 mm, cut with glass cutter) were covered with pieces of parafilm, where holes of ~ 5 mm diameter were punched in with hole puncher. To ensure adhesion between a parafilm and a substrate, the substrates with pieces of parafilm above were heated on a hotplate at 70-80 °C for 30-60 seconds, making parafilm partially melt and adhere to the surface of substrate.

**Preparation of the assay step by step**

1 Delivering of 30 μL solution of 40 μg/ml anti-human IgG antibodies dissolved in Phosphate Buffered Saline (PBS, pH=7.40) on each address for 4 hours in wet mini chamber (Figure S2, A)

R1. First Rinsing with 0.1% Tween 20 in PBS solution, 1+1 =2 mL for each address. (Figure S2, B)

2 .Next, 30 μL solution of Casein Block buffer was delivered on each address, left for 3 hours in wet chamber

R2 Recurrent Rinsing with 0.1% Tween 20 PBS solution (PBS-T) 1+1 mL for each address

3. 30 μL solution of antigen (analyte) human IgG in PBS solution delivered on the surface of each address and left for 4 hours in wet chamber

R3 Recurrent Rinsing with PBS-T solution 1+1 mL for each address

4. 30 μL solution/suspension of ERLs delivered on the surface of each address and left for 8 hours in wet chamber

R4 Final Rinsing with PBS-T solution 1+1 mL for each address

Sample drying → Ready for measurements

Preparation of ERLs (Extrinsic Raman Labels)

1. Add 10 μL of 1 mM solution of 4-NBT in acetonitrile and 50 μL of 50mM borate (pH=8.4) to 1.00 mL of 60 nm diameter suspension of gold nanoparticles in PBS in each of several micro-centrifuge tube and shake it for 2 h

2. Centrifuge the tubes with suspension at 2500 g for 5 minutes; remove supernatant (~0.95-0.95 mL) without disturbing gold nanoparticle at the bottom of each tube

3. Add 1.00 mL of 2mM Borate Buffer + 140 μL of 250 μg/mL solution of capture antibody (anti-human IgG) and shake for 6 hours

4. Add solution of BSA ( 120 μL of 10% Bovine Serum Albumin) to the tube and continue to shake the tube for another 5 hours

5. Centrifuge the tubes for 8 min at 2500 g, remove supernatant and add 1 mL of 1% BSA solution in 2mM borate buffer

6. Repeat the previous step ($2^{nd}$ cycle)

7. Repeat the previous step but finally add 300 mL of 1% BSA solution in 2mM borate buffer and 30 μL of 10% solution of NaCl to adjust ionic strength of the suspension close to the one of human blood.

8. Combine ERLs from several 4 tubes into one tube and filtrate the suspension through 0.2 μm pore diameter sterile syringe filter (4 mm) from Corning

9. Apply ERL suspension to the substrate for 8 hours = Step 4 of Substrate preparation

10. Rinse substrates twice with PBS for the last time

11. Wait until samples dry and do Raman measurements

After Raman measurements described in the manuscript, fluorescence background is corrected when average background ( in the 10 $cm^{-1}$ ranges left and right of the peak ) subtracted from Raman Intensity : BG- corrected Raman intensity is found. It is illustrated on Figure S2 C.

Table S1 SERS Data for calculation of LOD from logarithmic plots in MPT64 assays on Al foil and Gold film

| | | Blank Adjusted Raman Intensity ( BARI) , cps | | | | | | | |
|---|---|---|---|---|---|---|---|---|---|
| [MPT64], ng/ml | log[MPT] | Al foil, 633 nm | | Au Film, 633 nm | | Al foil, 785nm | | Gold film, 785nm | |
| 3.1 | 0.491 | 129 | | 222 | | 96 | | 762 | |
| 10 | 1.000 | 449 | | 485 | | 262 | | 1310 | |
| 31.6 | 1.500 | 872 | | 1290 | | 519 | | 2651 | |
| 300 | 2.477 | 1338 | | 1895 | | 816 | | 3939 | |
| | laser | slope | intercept | blank, cps | std blank | 3 std | log LOD | LOD, ng/ml | $R^2$ |
| Al foil | 633 nm | 614.9 | -143.4 | 203.1 | 10.2 | 30.5 | 0.28 | **1.9** | 0.986 |
| Al foil | 785 nm | 369.1 | -81.465 | 129.1 | 2.7 | 8.2 | 0.24 | **1.8** | 0.989 |
| Gold film | 633 nm | 883.9 | -235.38 | 760.8 | 56.0 | 167.9 | 0.46 | **2.9** | 0.960 |
| Gold film | 785 nm | 1662.1 | -106.85 | 1805.6 | 33.9 | 101.8 | 0.13 | **1.3** | 0.977 |

Table S2 SERS assay data for calculation of LOD from logarithmic plots in hIgG assays on Al foil and Gold film

| hIgG C, pM | log C, pM | Al foil | | Gold Film | | | | |
|---|---|---|---|---|---|---|---|---|
| | | BARI, counts | raw RI, counts | BARI, counts | Raw RI, cpounts | | | |
| blank | | 0 | 31.67 | 0 | 74.06 | | | |
| 30 | 1.4771213 | 14.43 | 46.10 | 22.8 | 96.86 | | | |
| 100 | 2 | 32.99 | 64.66 | 108.1 | 182.16 | | | |
| 300 | 2.4771213 | 69.49 | 101.16 | 364 | 438.06 | | | |
| 1000 | 3 | 90.72 | 122.39 | 613.2 | 687.26 | | | |
| 4000 | 3.60206 | 176.70 | 208.37 | 749.8 | 823.86 | | | |
| 10000 | 4 | 203.24 | 234.91 | 836.5 | 910.56 | | | |
| | Range, pM | intercept | slope | Std Blank | 3*std | log LOD | LOD | $R^2$ |
| Al foil | 30 - 1000 | -65.523 | 52.459 | 3.43 | 11.79 | 1.47 | 29.8 | 0.98 |
| Au film | 30 -1000 | -620.58 | 400.97 | 3.01 | 9.04 | 1.57 | 37.2 | 0.96 |

Data for assay on gold film is taken from Supplementary information in Bukasov et al publication. Spectra used for calculation of BARI ( ) of hIG assay on Al foil are shown on Figure 4 of this paper.

Table S3 Selectivity Assay Data or Non-specific signal of rat IgG vs specific signal of human IgG

| | Raw Raman Intensity | | | Blank Ajusted RI | | BABNRI | | |
|---|---|---|---|---|---|---|---|---|
| | blank | human | rat | human | rat BARI | human | rat | rat/human |
| Au 500 pM | 14.7 | 24.6 | 16.5 | 9.9 | 1.8 | 103% | 13% | 12.6% |
| Au 2000 pM | | 101.9 | 24.0 | 87.2 | 9.3 | 703% | 69% | 9.7% |
| Al 500 pM | 10.1 | 29.5 | 12.7 | 19.4 | 2.6 | 253% | 25% | 9.9% |
| Al 2000 pM | | 65.1 | 14.9 | 55.0 | 4.8 | 616% | 47% | 7.6% |
| Si 500 pM | 5.5 | 9.6 | 5.5 | 4.1 | 0.0 | 107% | 1% | 0.7% |
| Si 2000 pM | | 13.4 | 5.7 | 7.8 | 0.2 | 207% | 4% | 1.9% |

BABNRI is Blank Adjusted Blank Normalised Raman Intensity

**Another sandwich immunoassay of human, rat and rabbit IgG with SERS detection to compare selectivity on Gold, aluminum and silicon substrates.**

In order to probe selectivity (specificity) of hIgG SERS immunoassay, we also did a comparative SERS immunoassay of human and rat IgG on gold, Al foil, and silicon substrates modified with anti-human IgG capture antibody using 60 nm dimeter commercial gold nanoparticles ( suspension in PBS from Sigma-Aldrich). The results are reported on Figure S3 below. This figure shows Raman signals, obtained with 633 nm laser excitation before and after blank adjustments as well as Relative Nonspecific Blank Adjusted Raman Intensity (RNBAI).

According to the insert table on this figure S3 , assay specificity on Al foil relative to specificity on gold is 1.5-2.2 times higher (x1.8 average). For instance for the same 0.04 nM rabbit IgG concentration relative non-specific response on Al was 3.8% on Al and 8.6% on gold. However, specificity on Si is about 18 times higher since relative non-specific response of
0.5 nM rabbit IgG was 0.5% on Si and 9.0% on gold

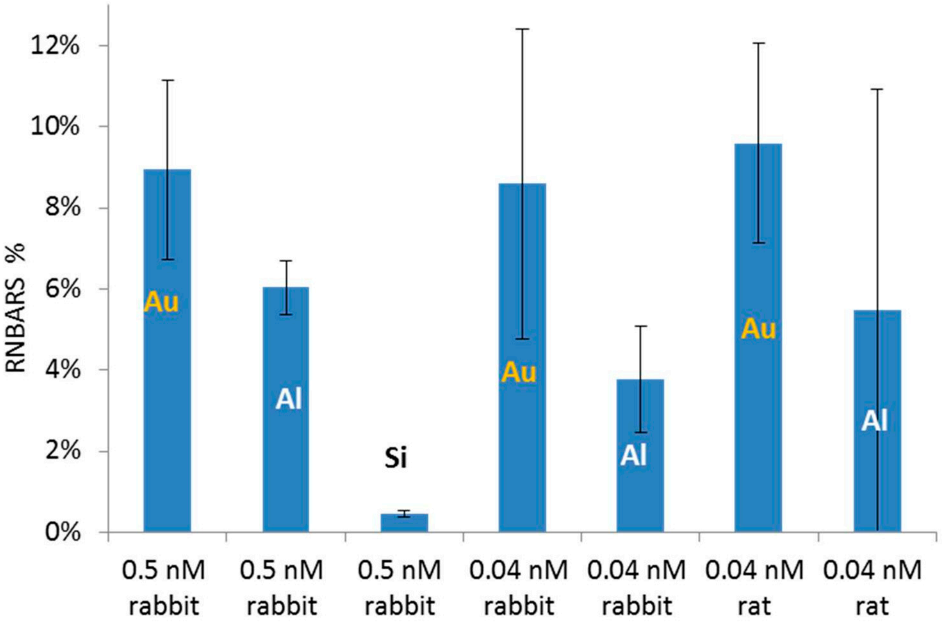


| [Antigen], nM | 0.5 | | | 0.04 | | 0.04 | |
|---|---|---|---|---|---|---|---|
| Substrate | Au | Al | Si | Au | Al | Au | Al |
| Non specific Antigen | rabbit IgG | | | | | rat IgG | |
| Raman Intensity, cps | 43.9 | 15.8 | 7.1 | 32.9 | 13.2 | 34.4 | 13.5 |
| BA Raman Intensity, cps | 24.2 | 3.54 | 0.23 | 13.2 | 0.88 | 14.7 | 1.3 |
| Specific antigen | human IgG | | | | | | |
| Raman Intensity, cps | 289.7 | 70.8 | 56.2 | 172.7 | 35.5 | 172.7 | 35.5 |
| BA Raman Intensity, cps | 270 | 58.5 | 49.4 | 153 | 23.2 | 153 | 23.2 |
| RNBARI, % | 9.0% | 6.1% | 0.5% | 8.6% | 3.8% | 9.6% | 5.5% |

*0.04 nM Rabbit IgG on Si is not shown because blank adjusted signal (o.o8 cps) is too low (uncertain) , BA is Blank Adjusted, RNBARI is relative normalized background adjusted Raman intensity

**Figure S3** Specificity/ selectivity in the SERS sandwich immunoassays for hIgG: three substrate comparison (gold, Al foil, silicon) of Relative Nonspecific blank Adjusted Raman Intensity (RNBARI).

The error bars of RNBARI are propagated from relative errors in blank and from relative error in non-specific signal (rat or rabbit IgG) , which depending on the substrate is about 10—200 times less than specific ( human IgG) signal . In fact relatively high error bars correlate with relatively small non-specific binding.

The This figure shows Raman signals before and after blank adjustments as well as Relative Nonspecific Blank Adjusted Raman Intensity (RNBAI). This assay is done with 60 nm gold nanoparticles used in preparation of ERLs

The insert table in the Figure S3 shows that the absolute non-specific responses or blank adjusted Raman intensities on gold are about order of magnitude (x11) higher on gold ( by factors of 24.2/3.54 = **7**;13.2/0.88 =**15** and 14.7/1.3=**12**) than on Al foil. For Au vs Si this contrast is even higher (24/0.23).

However, in order to get a true assessment of selectivity we need to compare the responses in relative terms and for that purpose RNBAIs were calculated. The RNBAI is a ratio (%) of non-specific blank adjusted Raman intensity to the specific blank adjusted Raman intensity or the ratio of the response to rabbit or response to rat in the numerator to the response to hIgG (specific) in the denominator for the particular antigen concentration.

For instance, for RNBAI for 0.5 nM of rabbit IgG on gold is 9.0% and it is calculated as 24.2 cps/270 cps. In this assay, all other assay parameters were identical for all antigens and all substrates.

The ratios of non-specific response on gold to non-specific response on Al foil are 9.0%/6.1%, 8.6%/3.8%, 9.6%/5.5%, when responses of 0.5 nM rabbit, 0.04 nM rabbit, 0.04 nM rat IgG are compared, respectively. When 0.5 nM rabbit IgG is tested as a non-specific antigen, the relative non-specific response on silicon shows is much smaller (0.5%) relive to relative non-specific response on gold ( 9.0% ) and on Al foil (6.1%).

Unfortunately for comparative calculations, but fortunately for potential practical applications, non-specific response for lower concentration (0.04 nM) of rabbit IgG on silicon is so low (0.08 cps), that it is within experimental uncertainty and therefore it cannot be used for comparison.

According to the insert table on this figure S4 , assay specificity on Al foil relative to specificity on gold is 1.5-2.2 times higher (x1.8 average). For instance for the same 0.04 nM rabbit IgG concentration relative non-specific response on Al was 3.8% on Al and 8.6% on gold. However, specificity on Si is about 18 times higher since relative non-specific response of
0.5 nM rabbit IgG was 0.5% on Si and 9.0% on gold

Overall this selectivity assay confirms the decrease in non-specific binding of both rat and rabbit IgG or in other words improvement in selectivity from gold film to Al foil and even more to silicon wafer as substrates for the simultaneous sandwich SERS immunoassays with identical assay parameters.

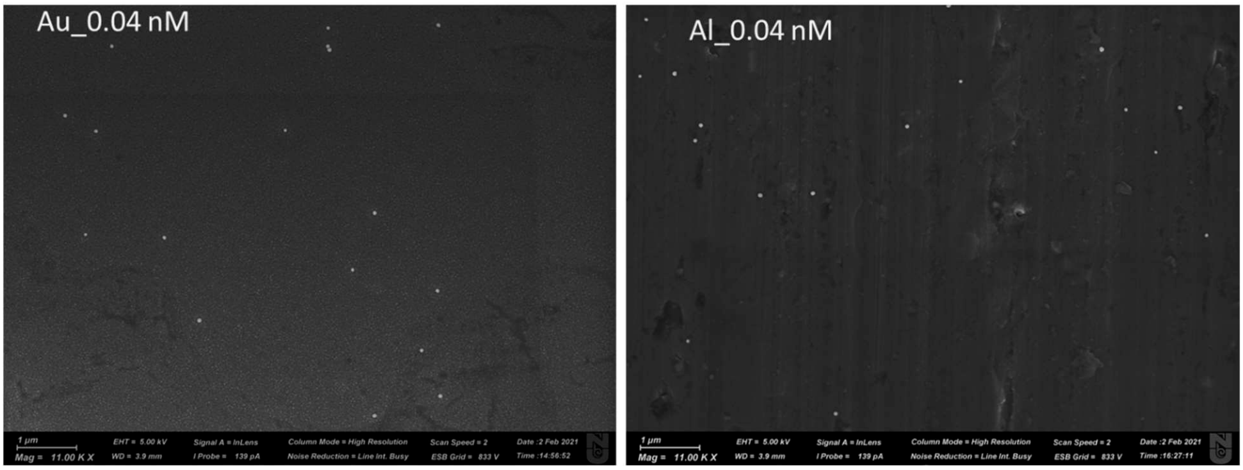


Figure S4 Representative SEM images for 0.04 nM human IgG samples on gold and on Al tape. 50 nm gold nanoparticles are used for ERL preparation.

Reference

1. Kunushpayeva, Z.; Rapikov, A.; Akhmetova, A.; Sultangaziyev, A.; Dossym, D.; Bukasov, R., Sandwich SERS immunoassay of human immunoglobulin on silicon wafer compared to traditional SERS substrate, gold film. Sensing and Bio-Sensing Research 2020, 29, 100355.
